# Real-time cardiovascular magnetic resonance at 1.5 T using balanced SSFP and 40 ms resolution

**DOI:** 10.1186/1532-429X-15-79

**Published:** 2013-09-12

**Authors:** Dirk Voit, Shuo Zhang, Christina Unterberg-Buchwald, Jan M Sohns, Joachim Lotz, Jens Frahm

**Affiliations:** 1 Biomedizinische NMR Forschungs GmbH am Max-Planck-Institut für biophysikalische Chemie, 37070, Göttingen, Germany; 2 Kardiologie und Pneumologie, Universitätsmedizin Göttingen, 37075, Göttingen, Germany; 3 Diagnostische und Interventionelle Radiologie, Universitätsmedizin Göttingen, 37075Göttingen, Germany; 4 DZHK (German Cardiovascular Research Center), partner site Göttingen, Göttingen, Germany

**Keywords:** Real-time CMR, Cardiovascular magnetic resonance, SSFP, Arrhythmias, Abnormal wall motion

## Abstract

**Background:**

While cardiovascular magnetic resonance (CMR) commonly employs ECG-synchronized cine acquisitions with balanced steady-state free precession (SSFP) contrast at 1.5 T, recent developments at 3 T demonstrate significant potential for T1-weighted real-time imaging at high spatiotemporal resolution using undersampled radial FLASH. The purpose of this work was to combine both ideas and to evaluate a corresponding real-time CMR method at 1.5 T with SSFP contrast.

**Methods:**

Radial gradient-echo sequences with fully balanced gradients and at least 15-fold undersampling were implemented on two CMR systems with different gradient performance. Image reconstruction by regularized nonlinear inversion (NLINV) was performed offline and resulted in real-time SSFP CMR images at a nominal resolution of 1.8 mm and with acquisition times of 40 ms.

**Results:**

Studies of healthy subjects demonstrated technical feasibility in terms of robustness and general image quality. Clinical applicability with access to quantitative evaluations (e.g., ejection fraction) was confirmed by preliminary applications to 27 patients with typical indications for CMR including arrhythmias and abnormal wall motion. Real-time image quality was slightly lower than for cine SSFP recordings, but considered diagnostic in all cases.

**Conclusions:**

Extending conventional cine approaches, real-time radial SSFP CMR with NLINV reconstruction provides access to individual cardiac cycles and allows for studies of patients with irregular heartbeat.

## Background

State-of-the-art cardiovascular magnetic resonance (CMR) relies on ECG-synchronized cine acquisitions with balanced SSFP contrast, typically at a magnetic field strength of 1.5 T [1-3]. On the other hand, recent work at 3 T reports significant potential for real-time CMR of cardiac function [4] and cardiovascular flow [5,6] at high spatiotemporal resolution usingT1-weighted radial FLASH with pronounced undersampling and image reconstruction by regularized nonlinear inversion (NLINV) [7,8]. This is because real-time SSFP CMR at 3 T is frequently affected by off-resonance “banding” artifacts due to magnetic field inhomogeneities and further limited by SAR regulations when trying to achieve sufficiently high flip angles for repetition times as short as 3 ms. In order to expand real-time CMR applications to a wider clinical community, this work combines the aforementioned acquisition and reconstruction advances with SSFP CMR at 1.5 T. Real-time CMR not only improves patient compliance because of free breathing and eventually shorter examination times, it also offers extended diagnostic opportunities by providing functional information about individual cardiac cycles and access to immediate physiologic responses to stress and exercise.

The proposed method employs a highly undersampled radial gradient-echo CMR technique with fully balanced gradients [9] in conjunction with serial image reconstruction by NLINV [7]. Extending previous studies by others [10-12], the method is directly applicable without the need for any calibration scan or averaging of multiple k-space data over time to obtain coil sensitivity maps for image reconstruction. This is because the underlying principle relies on an advanced parallel imaging method [13] that jointly estimates the image and all coil sensitivities from the undersampled dataset of a single frame in a self-consistent manner and thereby optimally exploits the available limited information.

The purpose of this work was twofold: First, to demonstrate the technical feasibility and achievable image quality of real-time radial SSFP CMR with NLINV reconstruction using studies of healthy subjects on two different 1.5 T CMR systems, and second, to evaluate the future clinical potential using preliminary applications to patients with cardiac arrhythmias and abnormal wall motion.

## Methods

### Subjects

Six participants with no known illness were recruited among the students of the local university. All subjects gave written informed consent before each CMR examination. A total of 27 consecutive patients (19 male, 8 female, age range 16–82 years, mean 54.0 ± 19.8 years) scheduled for cardiac diagnostics (mainly ischemia, function and scar) underwent additional real-time CMR. Respective movies along all relevant anatomic orientations were recorded as a final adjunct to the standard examination protocol lasting for a total duration of about 3 min. Informed consent was obtained from all patients. The local ethics board Ethikkommission Universitätsmedizin Göttingen approved the study.

### Undersampled radial gradient-echo CMR with balanced gradients

All acquisitions were based on a gradient-echo CMR sequence with radial encoding and fully balanced gradients as previously described [9]. The design results in a zero gradient moment (zero phase) for each repetition interval *TR* and a symmetric echo at *TE = TR*/2. The actual implementations employed a highly undersampled radial encoding scheme (i.e., 13 to 15 spokes per frame) where 5 successive acquisitions comprised complementary sets of spokes. The spokes of each frame were equally distributed over a full 360° circle in order to homogeneously sample k space. NLINV reconstructions [7] benefit from complementary sets of spokes by temporal regularization to the preceding frame, which constrains the range of possible solutions to the inverse problem. Moreover, the concept forces residual streakings to “flicker” from frame to frame which facilitates their removal by a post-processing temporal median filter.

Image reconstruction by NLINV was performed offline. Online monitoring at the same frame rate was accomplished by a sliding-window technique that relied on gridding and inverse FFT of a composite dataset of 5 consecutive radial acquisitions (i.e., 65 to 75 spokes) followed by subsequent shifts of the composite dataset by 1/5 [9]. Offline reconstructions of serial images from the highly undersampled single-frame acquisitions employed a parallelized algorithm of the NLINV method [14]. It was implemented on a computer equipped with 8 graphical processing units (GPU) and a specially designed library of newly developed basis functions for the efficient computing on multiple GPUs [15]. The current version achieves a reconstruction speed of about 20 frames per second.

### Real-time SSFP CMR

The real-time SSFP CMR sequence with radial undersampling was implemented on two commercial 1.5 T CMR systems (Siemens Healthcare, Erlangen, Germany). They were equipped with different gradient systems and used for studying healthy subjects (1.5 T Avanto) and patients (1.5 T TIM Symphony), respectively. In either case, radiofrequency excitation was accomplished with a body coil, while signal reception employed a multi-channel coil arrangement consisting of an anterior 8-coil array and two or three four-coil arrays from the spine coil (automatically selected depending on image position and orientation).

Details of the real-time SSFP CMR sequences are summarized in Table 1. The acquisitions resulted in serial images with a nominal resolution of 1.8 × 1.8 mm^2^ and 6 or 8 mm section thickness. The flip angle was set to a maximum possible value of 35° to comply with SAR regulations. The images covered a 256 × 256 mm^2^ FOV with a base resolution of 144 data samples (twofold oversampling) and 13 or 15 radial spokes. These parameters were chosen as the result of many preceding applications including versions with larger undersampling factors, higher spatial resolution or larger FOVs. In fact, a larger FOV remains an option which does not necessarily compromises the spatial and temporal resolution. For example, because of the high bandwidths used for real-time CMR, a 320 × 320 mm^2^ FOV with a base resolution of 176 data samples may be acquired with the same number of spokes (i.e., a higher but tolerable degree of undersampling) and an only slightly longer acquisition window which does not prolong the overall *TR*.

**Table 1:** Acquisition parameters for real-time SSFP CMR

	**CMR I**	**CMR II**
Imaging time/ms	41	40
Frame rate/s^-1^	25	25
Maximum gradient/mT m^-1^	40	30
Slew rate/mT m^-1^ ms^-1^	200	125
Resolution/mm^3^	1.8 × 1.8 × 6.0	1.8 × 1.8 × 8.0
Field-of-view/mm^2^	256 × 256	256 × 256
Base resolution	144	144
Acquired spokes	15	13
Nyquist undersampling factor	15	17.4
Repetition time/ms	2.72	3.08
Echo time/ms	1.36	1.54
Flip angle/degree	35	35
Bandwidth/Hz Pixel^-1^	1930	1020

Real-time CMR movies with a typical duration of 15 s (i.e., 12 to 15 heartbeats) covered the heart in sequential scans along anatomically defined orientations including three short-axis views (base, midcardial, apical) as well as a two-chamber, three-chamber, and four-chamber view.

### Cine SSFP CMR

Patient studies with conventional ECG-synchronized cine SSFP CMR (Cartesian encoding) and breath-holding conditions involved multiple views at 1.5 to 1.8 mm resolution and 6 or 7 mm section thickness. The flip angle was 60°, the repetition time 40 ms and the bandwidth 930 Hz pixel^-1^. The FOVs of 380 × 309 mm^2^ to 492 × 400 mm^2^ were generally larger than necessary for real-time radial SSFP CMR to avoid aliasing. The magnetic field homogeneity (product shim) was neither optimized for cine nor real-time SSFP CMR.

### Evaluations

Image quality was assessed by visual inspection of real-time and cine SSFP CMR movies by two blinded observers with 3 years (JMS) and 8 years (CU) experience of CMR evaluation, respectively. Scans were evaluated according to an image quality score ranging from 0 = no diagnostic quality to 1 = reduced diagnostic quality, 2 = many artifacts, 3 = some artifacts, and 4 = optimal diagnostic quality. Artifacts that were considered to affect the image quality primarily referred to SSFP banding artifacts. Cine CMR showed additional problems due to involuntary motions and real-time CMR presented with residual streakings.

Functional analyses were done by a team of experienced radiologists and cardiologists. A quantitative analysis of cardiac mass, chamber volumes and ejection fraction was achieved with the use of a standard software package (Medis Qmass, Leiden, The Netherlands) applied to each individual cardiac cycle separately. For real-time CMR the analyses were restricted to 5 consecutive heartbeats, while cine breath-hold CMR typically averaged data of 10–12 cardiac cycles. Comparative evaluations for real-time and cine SSFP CMR are reported for 19 out of 27 patients where a sufficient number of similar views were available. Nevertheless, residual differences in section alignment are unavoidable due to different positions during free breathing (real-time CMR) and breath-holding (cine CMR). Patients with normal chamber anatomy were analyzed using long-axis views (four-chamber and vertical long axis), while short-axis views served for the evaluation of patients with abnormal wall motion.

## Results

**Figure 1:**
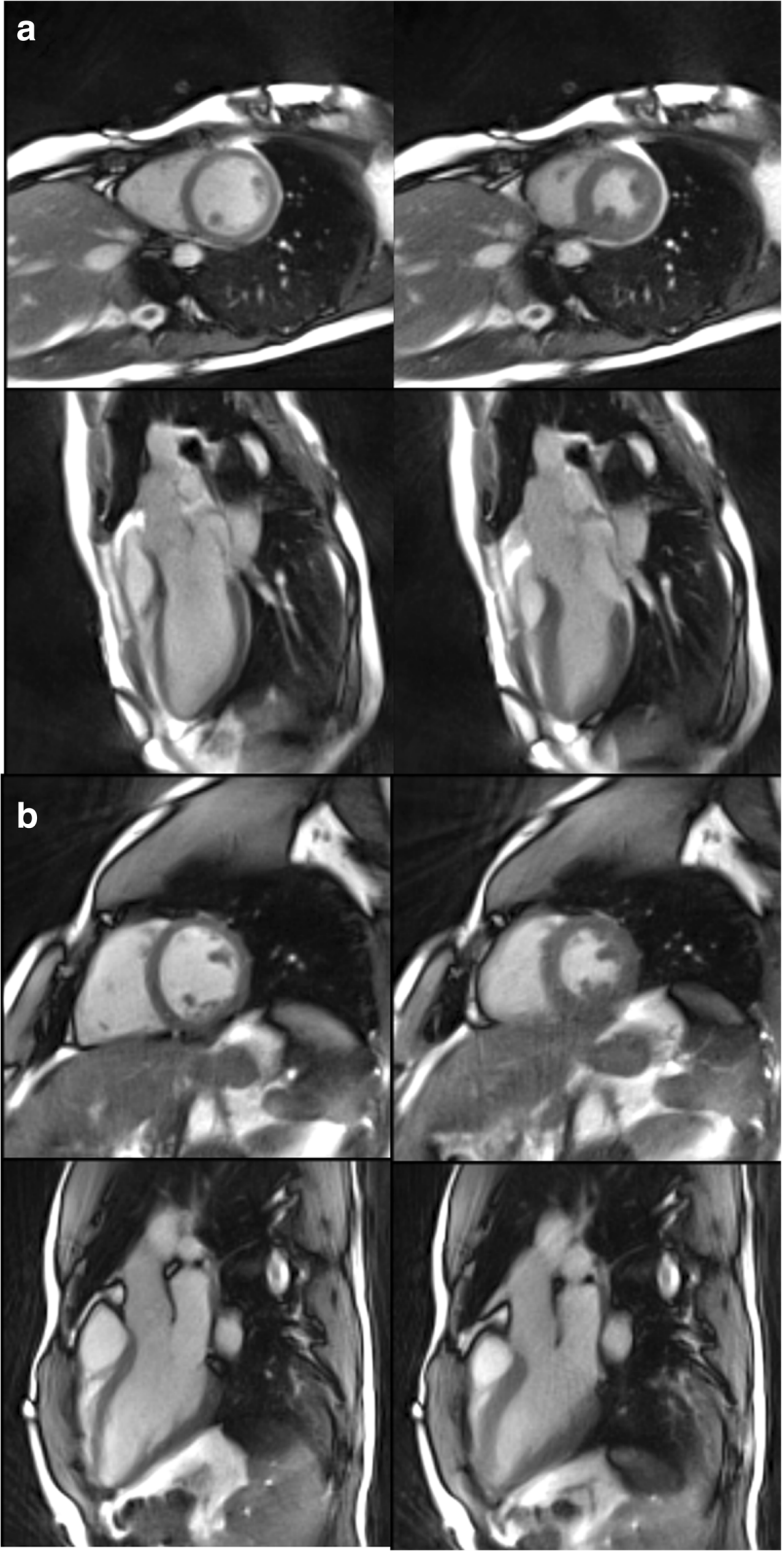
**Real-time SSFP CMR.** Short-axis and three-chamber views of two healthy subjects at (left) diastole and (right) systole. The images refer to selected frames of real-time CMR movies acquired with the use of **(a)** CMR system I at 41 ms resolution (TR/TE = 2.72/1.36 ms, 15 spokes) and **(b)** CMR system II at 40 ms resolution (TR/TE = 3.08/1.54 ms, 13 spokes). For other details see Table 1.

Figure 1 shows selected frames at peak systole and late diastole from real-time radial SSFP movies of the heart of a healthy subject and an asymptomatic patient. They were obtained using two different 1.5 T CMR systems with normal and enhanced gradient performance (see Table 1). The images in Figure 1a benefit from shorter *TR/TE* values that minimize the sensitivity of the SSFP CMR signal to “banding” artifacts and also allow for the acquisition of 15 spokes per 41 ms image. On the other hand, Figure 1b demonstrates that adequate image quality may equally well be obtained with the use of a more standard 1.5 T CMR system and only 13 spokes for a 40 ms acquisition. With respect to the Nyquist theorem, a fully sampled radial acquisition requires the number of spokes to equal the matrix resolution times π/2 (i.e., 144 data samples × 1.57 = 226 spokes). Thus, the degrees of undersampling used here (i.e., 13 and 15 spokes per frame) correspond to factors of 17 and 15, respectively. It should be noted that the signal from the myocardium appears slightly brighter than in conventional cine SSFP recordings because of a lower flip angle of 35° which is limited by SAR regulations at short *TR*. Nevertheless, the achievable contrast between the myocardial wall, blood and lung turns out to be fully sufficient for clinical use.

Table 2 summarizes the mean image quality scores obtained for real-time and cine SSFP image series in all 6 views. The values for cine recordings are slightly higher than for real-time CMR yielding 3.70 ± 0.58 and 3.33 ± 0.77 on average, respectively. In particular, real-time sequences had a lower performance in multi-chamber views and apical short-axis views, while the quality of short-axis views at base and midcardial position was almost comparable in both techniques. In fact, the real-time data were considered to be of diagnostic quality in all cases with a mean image quality score that translates into “good image quality with some artifacts”. Noteworthy, the three patients with arrhythmias (total of 18 views) presented with much better quality in real-time CMR (3.83 ± 0.38) than in cine CMR (2.83 ± 0.71).

**Table 2:** Image quality scores for real-time and cine SSFP CMR of patients

**Anatomical views**	**Number of views**^**#**^	**Real-time CMR**	**Cine CMR**
Total	306	3.33 ± 0.77	3.70 ± 0.58
Four-chamber view	49	3.25 ± 0.67	3.66 ± 0.69
Three-chamber view	48	3.20 ± 0.72	3.73 ± 0.54
Two-chamber view	51	3.21 ± 0.88	3.71 ± 0.67
Short-axis base	53	3.67 ± 0.51	3.85 ± 0.43
Short-axis midcardial	53	3.50 ± 0.64	3.81 ± 0.48
Short-axis apex	52	3.10 ± 0.98	3.56 ± 0.64

Values are given as mean ± standard deviation: 0 = no diagnostic quality, 1 = reduced diagnostic quality, 2 = many artifacts, 3 = some artifacts, 4 = optimal diagnostic quality.

^#^Only those views of the 27 patients were included that were available for both sequences, unpaired views were excluded.

**Figure 2:**
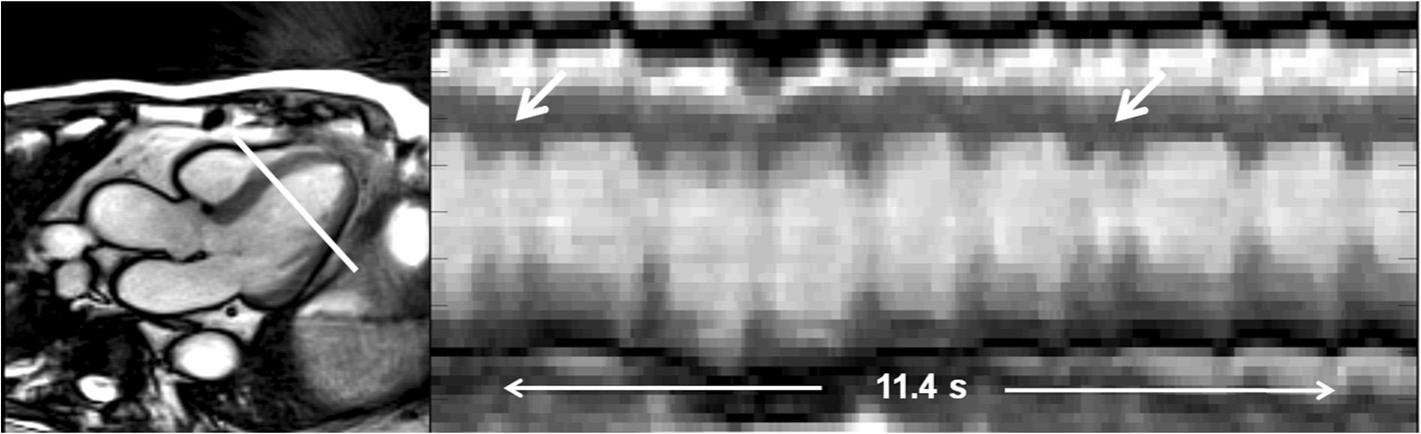
**Arrhythmias.** Real-time SSFP CMR of a patient with supraventricular arrhythmias at 40 ms resolution (for details see Table 1). (Left) Selected diastolic frame of a three-chamber movie and (right) corresponding “1D arrhythmia plot” depicting the temporal profile of the indicated reference line. The diagram covers 10 normal contractions and identifies two atrial extrasystoles (285 images = 11.4 s). See also Additional files 1 and 2 for real-time CMR movies of the same patient in a short-axis and four-chamber view.

**Figure 3:**
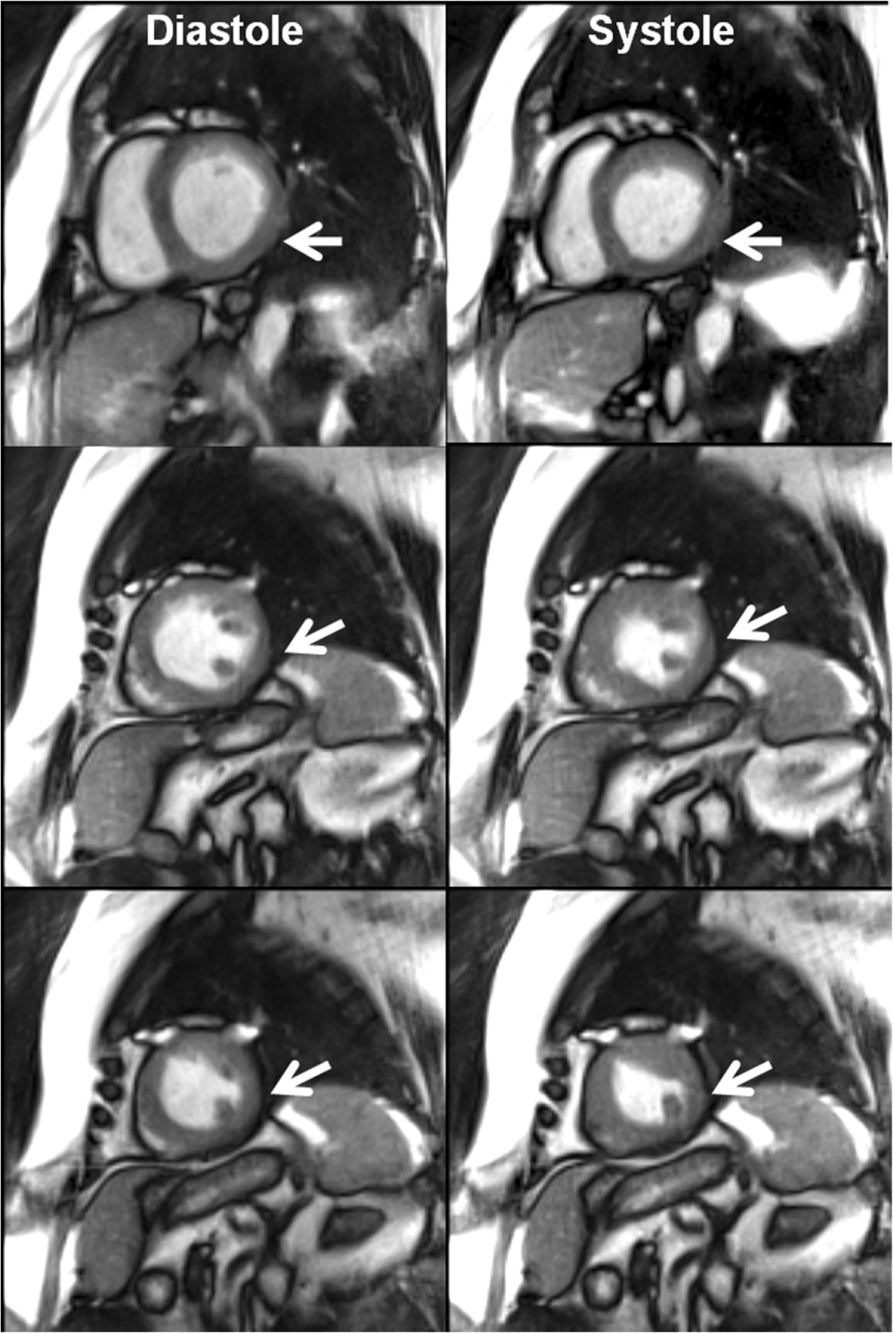
**Abnormal wall motion.** Real-time SSFP CMR of a patient with abnormal motion of the left-ventricular myocardial wall at 40 ms resolution (for details see Table 1). (Left) Selected diastolic and (right) systolic frames of three short-axis movies from (top) base to (bottom) apex visualize the reduced thickening and contraction of the myocardial wall (arrows). See also Additional files 3 and 4 for real-time CMR movies of the same patient in a short-axis and three-chamber view.

Selected clinical examples are presented in Figures 2 and 3 for two patients with supraventricular arrhythmias and abnormal wall motion, respectively. Of course, aperiodic movements of the heart pose no technical obstacle for real-time CMR recordings. For the patient in Figure 2 arrhythmic events are easily depicted in additional movies [see Additional files 1 and 2] each showing 10 normal contractions and two atrial extrasystoles in a short-axis and four-chamber view. Another way of visualizing the occurrence of arrhythmic events is demonstrated in Figure 2 where the temporal intensity profile taken along a reference line in a three-chamber movie may be considered a “1D arrhythmia plot”. Such diagrams largely facilitate the identification of extrasystolic beats and the evaluation of cardiac functions for selected cycles.

Figure 3 depicts diastolic and systolic frames selected from three short-axis movies of a patient with abnormal motion of the inferior and infero-lateral segments of the left-ventricular myocardial wall due to known myocardial infarction. The lack or severe reduction of wall thickening and contraction is well depicted in all three sections though less pronounced in the basal plane and more clearly in the two lower sections closer to the apex. These motion abnormalities are even more easily detected in corresponding real-time CMR movies [see Additional files 3 and 4] in a short-axis and three-chamber view, respectively.

Table 3 summarizes quantitative evaluations of myocardial mass, left-ventricular volumes, stroke volume and ejection fraction for both real-time and cine SSFP CMR of 19 patients where three comparable short-axis views were available. Statistically significant differences between real-time and cine parameters (paired *t* test) were only obtained for the ejection fraction yielding about 10% smaller values for real-time CMR. While the respective mean values represent averages across patients, the assessment of the individual beat-to-beat variation revealed rather stable conditions (± 5%) for real-time CMR despite free breathing.

**Table 3:** Evaluations of real-time and cine SSFP CMR (19 patients)

	**Real-time CMR free breathing**	**Beat-to-beat variation**	**Cine CMR breath-holding**
Myocardial mass/g	35.5 ± 10.5	± 4%	35.1 ± 10.5
Enddiastolic left-ventricular volume/ml	49.5 ± 13.9	± 3%	52.1 ± 18.2
Endsystolic left-ventricular volume/ml	26.7 ± 13.9	± 4%	24.4 ± 11.9
Stroke volume/ml	22.8 ± 5.2	± 7%	28.5 ± 15.7
Ejection fraction/%	49 ± 12^#^	± 5%	55 ± 14

Values (mean ± standard deviation) were obtained from three short-axis views and averaged across patients (cine CMR covering 10–12 heartbeats) and cardiac cycles (real-time CMR, 5 consecutive heartbeats, beat-to-beat variation).

^#^Statistically significant different from cine CMR (paired *t* test).

## Discussion

Cardiovascular imaging probably represents the most relevant field of application for real-time MRI. This is because examinations of myocardial anatomy, function, and blood flow during free breathing and independent of an ECG-synchronized data acquisition promise improved patient compliance, extended diagnostic capabilities, and temporal (economic) efficiency. The present work demonstrates that such applications may not only be performed at 3 T using T1-weighted FLASH [4-8], but also at a lower field strength of 1.5 T and with the use of the hitherto more “conventional” SSFP contrast. Real-time CMR acquisitions based on undersampled radial SSFP sequences may be implemented on existing CMR scanners without any hardware modification. On the other hand, online visualization of the NLINV reconstructions requires a parallelized version of the algorithm and a GPU-based computer bypassing the conventional reconstruction pipeline (see below).

Previous radial SSFP approaches to CMR predominantly aimed at a more efficient (i.e., accelerated) 3D coverage of the heart, e.g. see [16-19]. Conversely, radial SSFP CMR has only rarely been applied for cross-sectional real-time CMR [20-22]. In comparison to the present results, however, such studies were restricted to healthy volunteers and offered lower spatial resolution of ≥ 2.5 mm as well as much longer acquisition times of 100–300 ms, while high display rates in terms of frames per second could only be achieved by view sharing and sliding-window reconstruction. More recent attempts that provide similar high frames rates as achieved here are k-t BLAST and SENSE approaches with optimized sampling schemes [10], through-time radial GRAPPA techniques [11] or combinations based on k-t SPARSE-SENSE [12]. These techniques enhance the degree of undersampling of conventional parallel imaging by exploiting temporal information, but require either the acquisition of lengthy calibration scans prior to each real-time acquisition or extensive data averaging over time to obtain the coil sensitivity maps needed for image reconstruction. In contrast, this work describes the implementation and first application of a robust real-time SSFP CMR method which is truly self-consistent in the sense that no pre-scan or any other supplementary information is required for serial image reconstruction of individual frames.

In a technical sense, the current real-time images eventually suffered from a local and transient appearance of residual streaking artifacts which are due to imperfect estimations from highly undersampled data. It turns out that such problems mostly originate from the site of a pronounced SSFP artifact which, for example, may be due to air in the abdominal regions below the heart. This is because the SSFP problems usually involve signals with both high contrast and high spatial frequencies, which are particularly sensitive to extreme undersampling. This may also apply to small anatomical structures with bright intensities. Future applications will be able to further reduce residual streakings by taking advantage of receiver coils with a larger number of independent elements.

In general, none of the aforementioned problems resulted in a reduced diagnostic assessment of the left or right ventricle. In absolute terms, the achieved image quality of the real-time CMR movies was slightly lower than that of cine recordings – provided the latter were not affected by respiratory motions or irregular heartbeats. If such problems can be excluded, cine acquisitions that extend over several seconds must of course yield images with better SNR and spatial resolution than obtainable within 30 to 40 ms. Nevertheless, the quality of the real-time short-axis views at 1.8 mm nominal resolution turned out to be almost comparable to that of the cine studies at 1.5 mm resolution (see also Additional files 1, 2, 3 and 4). This is because the ability to readily identify wall motions and blood-myocardium borders may be more relevant for a clinical assessment than the mere spatial resolution. Thus, the contrast and temporal accuracy of the real-time images may lead to a similar diagnostic utility as obtained for cine images at slightly higher spatial resolution. Moreover, 1.5 T CMR systems with multi-element coils and faster gradient systems are expected to further improve the technical quality of real-time images.

Functional evaluations of the preliminary patient data resulted in quantitatively comparable results for real-time and cine CMR with the exception of a 10% lower ejection fraction (averaged across patients). While such differences are difficult to interpret in the absence of an *in vivo* gold standard, they may reflect systematic differences in image acquisition and physiological condition when trying to compare ECG-synchronized cine CMR with breath-holding, which merges data from 10–12 heartbeats, with real-time CMR of individual heartbeats during free breathing. The present findings suggest that real-time SSFP CMR will generally reach diagnostic quality, while being superior for patients with irregular heartbeats.

At this stage, practical limitations of the proposed method are mainly due to the high computational demand of the NLINV reconstruction. In order to fully exploit the real-time CMR capacity, i.e. to overcome the online sliding-window visualization, the NLINV algorithm requires a multi-GPU computer. Such a system is routinely working in our 3 T CMR system where it has been integrated into the software architecture as an “invisible” bypass computer without the need for any user interference [8]. However, it is not yet commercially available and so far has not been implemented into the 1.5 T CMR system used for patient studies. Another challenge is the need for adaptations of the existing post-processing software for parametric evaluations. In order to retain and extract information about pathophysiologic variations, such programs should be able to efficiently and reliably deal with several hundreds of images from multiple heartbeats rather than analyze 20–30 images from a single cardiac cycle. This particularly refers to a fully automated segmentation of the myocardium without the need for manual corrections.

## Conclusions

This work describes a robust acquisition and reconstruction technique for real-time CMR at 1.5 T using conventional SSFP contrast. The NLINV method is directly applicable and fully self-consistent using only data from a single frame without the need for any calibration scan or other computation of averaged coil sensitivity maps. The acquisition technique operates on commercial CMR systems without hardware modification, while rapid image reconstructions require a dedicated computer equipped with GPUs. Preliminary applications to patients with arrhythmias and abnormal wall motion promise considerable clinical potential. Real-time vs cine CMR validation studies of well-defined cohorts of patients are now warranted.

## Abbreviations

BLAST: Broad-use linear acquisition speed-up technique; ECG: Electrocardiogram; FFT: Fast Fourier transform; FLASH: Fast low-angle shot; FOV: Field of view; GPU: Graphical processing unit; GRAPPA: Generalized autocalibrating partially parallel acquisition; CMR: Cardiovascular magnetic resonance; NLINV: Regularized nonlinear inversion; SAR: Specific absorption rate; SENSE: Sensitivity encoded; SNR: Signal-to-noise ratio; SSFP: Steady-state free precession; *TE*: Echo time; *TR*: Repetition time.

## Competing interests

SZ and JF hold a patent about the real-time CMR acquisition and reconstruction technique as used here. All other authors declare that they have no competing interests.

## Authors’ contributions

DV helped to develop the CMR acquisition and reconstruction technique and in the interpretation of the results. SZ helped to design the study and to develop the CMR acquisition technique, and assisted in the interpretation of the results. CU helped to design the study, performed the clinical evaluations, and assisted in the interpretation of the results. JMS performed the clinical evaluations and assisted in the interpretation of the results. JL conceived the study and participated in its design, supervised the data acquisition, and interpreted the results. JF conceived the study and participated in its design, helped to interpret the results and drafted the manuscript. All authors read and approved the final manuscript.

## Supplementary Material

Additional file 1 **Real-time SSFP CMR of arrhythmias: Short-axis view.** Real-time SSFP CMR movie of a patient with supraventricular arrhythmias at 40 ms resolution (for details see Table 1). Click here for file

Additional file 2 **Real-time SSFP CMR of arrhythmias: Four-chamber view.** Real-time SSFP CMR movie (four-chamber view) of a patient with supraventricular arrhythmias at 40 ms resolution (for details see Table 1). Click here for file

Additional file 3 **Real-time SSFP CMR of abnormal wall motion: Short-axis view.** Real-time SSFP CMR movie of a patient with abnormal motion of the left-ventricular myocardial wall at 40 ms resolution (for details see Table 1). Click here for file

Additional file 4 **Real-time SSFP CMR of abnormal wall motion: Three-chamber view.** Real-time SSFP CMR movie of a patient with abnormal motion of the left-ventricular myocardial wall at 40 ms resolution (for details see Table 1). Click here for file
